# LCP-CAS: Lattice-Based Conditional Privacy-Preserving Certificateless Aggregation Signature Scheme for Industrial IoT

**DOI:** 10.3390/e28030258

**Published:** 2026-02-26

**Authors:** Lin Shi, Ziyi Chen, Ziyan Zhang, Pan Chen, Liquan Chen

**Affiliations:** School of Cyber Science and Engineering, Southeast University, Nanjing 210096, China; 230209338@seu.edu.cn (L.S.); 220245557@seu.edu.cn (Z.C.); amblyan@seu.edu.cn (Z.Z.); chenpan@seu.edu.cn (P.C.)

**Keywords:** certificateless aggregate signature, lattice-based, conditional privacy preservation, industrial Internet of Things

## Abstract

Aiming at the challenge that traditional signature schemes struggle to simultaneously achieve efficiency, resistance to quantum attacks, and privacy protection, this paper proposes a lattice-based conditional privacy-preserving certificateless aggregate signature method (LCP-CAS). The scheme adopts an unordered aggregation algorithm to compress multiple signatures, in arbitrary order, into a single fixed-length aggregate signature, thereby achieving linear scalability in verification complexity. Its security is based on the hardness of the Ring Short Integer Solution (RSIS) problem, ensuring post-quantum resistance. By incorporating a conditional privacy-preserving mechanism, the scheme realizes device anonymity while supporting identity traceability, thus balancing privacy protection with regulatory requirements. Security analysis shows that the scheme meets the security requirements, including integrity, non-repudiation, conditional privacy preservation, and resistance to collusion attacks. Compared with existing related schemes, LCP-CAS achieves reduces aggregation and verification overhead while maintaining practicality in large-scale settings such as industrial IoT and device monitoring.

## 1. Introduction

With the continuous convergence and advancement of technologies such as the Internet of Things (IoT), big data, and artificial intelligence (AI), the Industrial Internet of Things (IIoT) has become a key driver of the intelligent, digital, and green transformation of industrial systems [1,2]. IIoT connects diverse industrial sensors and smart devices to the internet, forming an interconnected intelligent network. However, the open and highly interconnected nature of IIoT also introduces unprecedented information security challenges [3], and ensuring data confidentiality, integrity, trustworthy identities, and tamper resistance has become a prerequisite for its sustainable development [4].

Digital signature technology, as a core mechanism for guaranteeing data integrity and identity authentication, plays an indispensable role in providing security for numerous critical application scenarios in industrial IoT [5,6,7]. In such environments, individual devices typically need to frequently exchange data with other devices or the cloud. If each device independently signs every outgoing data item, this leads to substantial communication and storage overhead. Consequently, the ability of aggregate signatures to compress multiple signatures into a compact form has prompted their widespread adoption in industrial IoT settings [8]. At the same time, the rapid development of quantum computing poses severe challenges to signature schemes based on traditional number-theoretic problems (such as the elliptic curve discrete logarithm problem) [9,10]. Designing signature schemes that are resistant to quantum computing attacks has therefore become an urgent problem in post-quantum cryptographic security research.

Quan et al. [11] proposed a lattice-based aggregate signature scheme that deeply integrates the core advantages of the CRYSTALS-Dilithium post-quantum digital signature algorithm with a zero-knowledge scalable transparent argument of knowledge protocol. By constructing an efficient signature aggregation mechanism, their scheme significantly improves Bitcoin transaction processing capability while preserving post-quantum security. In [12], a lattice-based Byzantine fault-tolerant aggregate signature scheme was proposed, achieving a notable breakthrough in the aggregate signature paradigm. Compared with existing schemes, this work introduces a flexible interaction mechanism that establishes a new balance between fully non-interactive and strongly interactive aggregate signatures: it retains the necessary interaction among signers while tolerating arbitrary Byzantine behavior by participants during the execution of the signature protocol (including node failures and malicious attacks). Lu et al. [13] presented a lattice-based disordered aggregate signature scheme that uses intersection operations to construct aggregate signatures, fundamentally mitigating the security risk that a single signer forges the participation of other signers in aggregation. However, due to the complex computational characteristics of lattice cryptography and the high cost of intersection algorithms, this scheme suffers from significant performance bottlenecks in signature generation and verification. Boneh et al. [14] proposed a one-time aggregate signature scheme, opening up a new research direction for aggregate signatures. Reference [15] proposed a secure and efficient certificateless aggregate signature authentication scheme with pseudonyms for vehicular ad hoc networks (VANETs). The scheme constructs a pairing-free elliptic-curve-based certificateless aggregate signature that combines time-varying pseudonyms with users’ secret values to achieve conditional privacy preservation while supporting efficient batch authentication in VANET environments.

An in-depth analysis of existing aggregate signature schemes reveals that, despite notable progress in efficiency, security, or functionality, several critical challenges remain unresolved. In particular, current schemes often fail to simultaneously satisfy the stringent requirements of industrial IoT environments—namely, high efficiency, strong security, and robust privacy protection. Some schemes prioritize communication efficiency but weaken key management security; others mitigate identity forgery risks at the cost of introducing severe performance bottlenecks. Identity-based schemes can offer post-quantum security when built upon quantum-resistant primitives; however, they inevitably incur the key escrow problem. These limitations substantially hinder the practical deployment of aggregate signatures in industrial IoT scenarios.

An in-depth review of existing aggregate signature schemes reveals that, while progress has been made in efficiency, security, or functionality individually, critical challenges remain unresolved—particularly in meeting the combined demands of industrial IoT environments: high efficiency, strong security, and robust privacy protection.

Existing schemes typically satisfy only a subset of these requirements due to the high cost of certain lattice operations (e.g., intersection-based aggregation or heavy polynomial multiplications), the key-escrow drawback of identity-based designs, or the added complexity introduced by privacy mechanisms. Some prioritize communication efficiency but compromise key management security. Others resist identity forgery at the cost of severe performance degradation. Identity-based constructions, even when instantiated with post-quantum primitives, remain inherently subject to the key escrow problem. These persistent limitations hinder the practical deployment of aggregate signatures in IIoT scenarios.

Based on existing research, this paper proposes an innovative lattice-based conditional privacy-preserving certificateless aggregate signature (LCP-CAS) scheme specifically designed for industrial IoT applications. The scheme addresses the limitations of current technologies through a multi-faceted design, achieving simultaneous optimization of efficiency, security, and privacy protection. Specifically, this paper makes the following three main contributions:

(1) We design the LCP-CAS scheme, which integrates a disordered aggregation algorithm, a conditional privacy-preserving mechanism, and lattice-based cryptography into a unified framework. This design not only guarantees quantum-resistant security but also significantly enhances privacy protection for industrial IoT systems.
(2) We provide a provable security analysis showing that LCP-CAS is secure against adaptive chosen-message attacks and collusion attacks by three types of adversaries (A1, A2, and A3) in the random oracle model. The scheme satisfies industrial security requirements, including integrity, non-repudiation, conditional privacy preservation, and forward secrecy.
(3) We implement LCP-CAS and conduct comparative experiments against four state-of-the-art lattice-based aggregate signature schemes. The results show that, for the aggregation of 100 signatures, the proposed scheme achieves an average aggregation time of 143 ms and a verification time of 232 ms, with verification efficiency improved by up to 30.7 times. Furthermore, the scheme maintains linear scalability with graceful performance degradation as the number of aggregated signatures increases, thereby meeting the real-time requirements of large-scale industrial IoT deployments.

## 2. Preliminaries

This section introduces the preliminary concepts required for the proposed scheme, including notation, hard lattice problems, the rejection sampling technique, and the definition of certificateless aggregate signatures.

### 2.1. Notations

In this paper, let n be a power-of-two integer, and q=1 mod 2n be a sufficiently large public prime modulus, with bounding parameter β satisfying β<q.

The notation ∥·∥ denotes the Euclidean norm (i.e., vector length). For a vector $x=\left(x_{1},x_{2},...,x_{n}\right),$ its Euclidean norm is defined as $∥x∥=\sqrt{x_{1}^{2}+x_{2}^{2}+\ldots +x_{n}^{2}}$. The infinity norm $∥\cdot {∥}_{\infty }$ is defined as: $\forall e\in Z_{q}^{m},∥e{∥}_{\infty }={max}_{i}^{m}\left|e_{i}\right|,$ which returns the maximum absolute value among all components of vector e.

We define a random distribution $X_{B}$ where $\forall x\in X_{B},∥x{∥}_{\infty }\leq B$, meaning any sample drawn from this distribution has an infinity norm bounded by B.

$Z^{m}$ denotes the m-dimensional integer vector space whose elements are vectors of m integers; $Z_{q}$ represents the prime finite field containing all integers in $\left(-q/2,q/2\right)$. A matrix $A\in Z_{q}^{n\times m}$ is formed by m uniformly random n-dimensional vectors $a_{i}\in Z_{q}^{n}$.

The polynomial ring $R_{n,q}=Z_{q}\left[x\right]/x^{n}+1$ consists of degree-(n−1) polynomials with coefficients in $\left[-\left(q-1\right)/2,\left(q-1\right)/2\right]$. $R_{n,k}$ denotes the subset of $R_{n,q}$ where polynomial coefficients are constrained to $\left[-k,k\right]$.

### 2.2. Hardness Assumption

The security of cryptographic schemes on lattices mainly relies on the hardness of several fundamental lattice problems. This subsection provides a detailed introduction to the hard problems on which the proposed scheme depends.

**Definition 1** (Ring Short Integer Solution, RSIS)**.** *Given a polynomial ring* $R_{q}$ *and a random vector* $a=\left(a_{1},a_{2},\ldots ,a_{m}\right)\in R_{q}^{m}$*, the RSIS problem requires finding a non-zero vector* $\nu \in R_{q}$ *with* $∥v{∥}_{\infty }\leq \beta$ *such that* aν=0(mod q).

The security of our scheme relies on the hardness of the RSIS problem.

**Definition 2** (Decisional Compact Knapsack problem, DCK)**.** *The DCK problem is to distinguish between:*

*1.*  *Uniform distribution over* $R_{q}\times R_{q}$*;*
*2.*  *The distribution* $\left(a,\;as_{1}+s_{2}\right)$*, where* a *is uniform in* $R_{q}$*, and* $s_{i}$* are uniform in* $R_{q,1}$

As summarized by Wang et al. [16], these problems, including SIS and its ring variant RSIS, enjoy worst-case to average-case reductions that ensure strong post-quantum resistance. Recent analyses further demonstrate that the DCK problem and its variants remain computationally infeasible under practical parameters [17].

In LCP-CAS, the unforgeability of an individual certificateless signature is mainly reduced to the DCK problem, while RSIS is used for the aggregate-signature forgery reduction.

### 2.3. Rejection Sampling

Rejection sampling has become a fundamental tool in lattice-based cryptography to ensure that signature outputs are statistically independent of secret keys. Comprehensive surveys describe its principle and implementation in discrete Gaussian samplers [18].

Given two probability distributions f and g, and a constant M∈R such that $f\left(x\right)\leq Mg\left(x\right)$ for all x, sampling z from g and outputting it with probability $f\left(z\right)/Mg\left(z\right)$ yields a result distributed according to f.

**Lemma 1**  [19]**.** *Let* V *be an arbitrary set,* h:V→R*,* $f:Z^{m}\to R$ *two probability distributions. If* $g_{v}:Z^{m}\to R$ *is a family of probability distributions indexed by* v∈V *satisfying:*

(1) $$\forall v\in V,\forall z\in Z^{m},M\cdot g_{v}\left(z\right)\geq f\left(z\right)$$

*then the statistical distance between the outputs of the following two algorithms is at most* $2^{-w\left(\log m\right)}/M$*:*

*1.*  *Sample* $v\leftarrow h,z\leftarrow g_{v}$*, then output* $\left(z,v\right)$ *with probability* $f\left(z\right)/M\cdot g_{v}\left(z\right)$
*2.*  *Sample* v←h,z←f*, then output* $\left(z,v\right)$ *with probability* 1/M

The rejection sampling algorithm based on the single-peaked Gaussian proposed by Lyubashevsky [19] is widely used. To further improve the efficiency of rejection sampling, Ducas et al. [20] proposed a rejection sampling algorithm based on a double-peaked Gaussian distribution. The double-peaked Gaussian uses the asymptotic square root of the security parameter as a coefficient, thereby reducing the standard deviation of the signature, shortening sampling time, and accelerating signature generation.

### 2.4. Certificateless Aggregate Signature

The concept of aggregate signatures allows multiple signers to combine distinct signatures into a single compact form, significantly reducing verification cost and communication overhead. Recent comprehensive surveys discuss the evolution of certificateless aggregate signatures and their applications in IoT and cloud environments [21].

Give *n* distinct messages independently signed by *n* users, a specialized algorithm can compress these individual signatures into a single compact aggregate signature. Verifiers need only inspect this aggregate signature alongside the original message set to validate the authenticity of all constituent signatures. Compared to traditional schemes, aggregate signatures offer two transformative benefits: (1) Efficiency: Aggregating multiple signatures into a single form enables the validity of all original signatures to be determined through a single verification process, significantly improving verification efficiency; (2) Security: Since the aggregation process incorporates the security features of multiple signatures, any forgery or tampering would require breaching the security protections of all original signatures, significantly enhancing the system’s overall security protection level.

**Figure 1:**
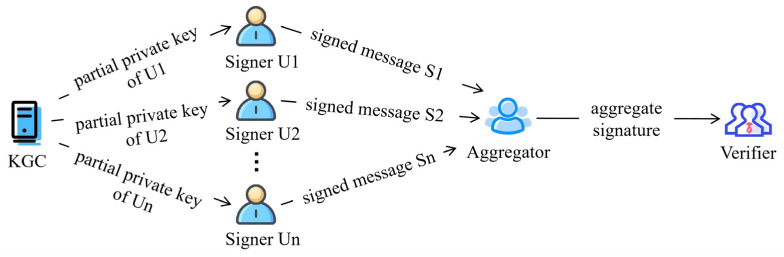
Certificateless aggregate signature model.

To fully leverage the advantages of certificateless signatures and aggregate signatures, researchers have extensively investigated certificateless aggregate signature (CLAS) schemes. As depicted in Figure 1, a canonical CLAS framework comprises four entities: the Key Generation Center (KGC), the signers, the aggregator, and the verifier.

## 3. System Model and Security Model

### 3.1. System Model

**Figure 2:**
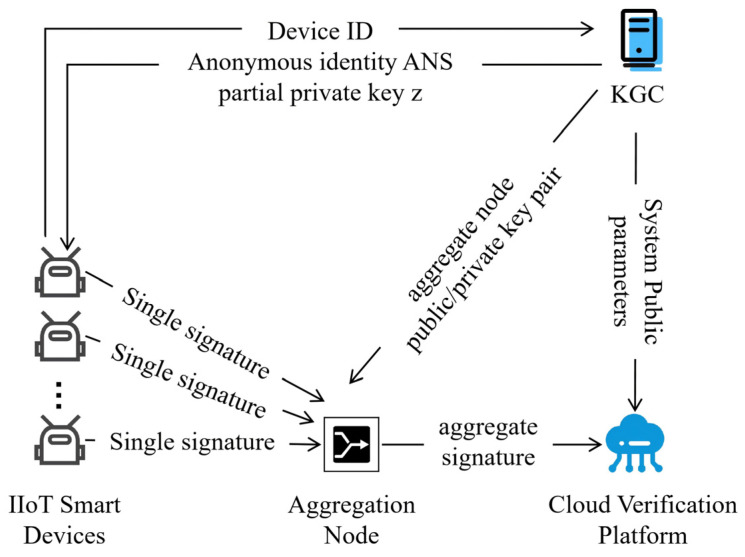
LCP-CAS system model.

As shown in Figure 2, the architecture of the proposed signature system consists of four fundamental components: the Key Generation Center (KGC), IIoT intelligent devices (IISD), aggregation nodes (AN), and the cloud verification platform (CVP).

Key Generation Center (KGC): The KGC is responsible for initializing global system parameters and generating partial private keys. Specifically, it first generates the system master key and public parameters. Then, for each IISD, it derives anonymous identity information based on the device ID and generates a partial private key bound to this anonymous identity. Subsequently, it delivers the above information to the corresponding device through a secure channel. Additionally, the KGC is also required to maintain a device identity list and support the key update function.

IIoT Intelligent Device (IISD): After receiving the partial private key distributed by the KGC, the IISD autonomously generates a secret value and combines it with the partial private key to generate the complete private key. The device uses the complete private key to generate certificateless signatures for collected industrial data (such as sensor readings and device status), ensuring the authenticity and integrity of the data. IISDs must also securely store secret values and periodically synchronize key status (e.g., update requests) with the KGC.

Aggregation Node (AN): The AN dynamically aggregates the signatures generated by different IISDs into a single compact signature according to their actual arrival order. During the signature aggregation process, the AN performs lightweight pre-verification: by quickly checking the basic validity of individual signatures (e.g., timestamp freshness, format compliance), it filters out abnormal signatures and prevents invalid signatures from entering the subsequent aggregation phase.

Cloud Verification Platform (CVP): The CVP is responsible for signature verification and global management. It verifies the validity of received signatures using system parameters publicly available from the KGC and the public key of the AN. The CVP must provide timestamping services to prevent replay attacks and record audit logs for traceability; after verification, it is responsible for processing and storing the large amounts of collected data.

### 3.2. Security Model

Certificateless aggregate signature schemes typically consider three distinct types of attackers [22]. In addition, in IIoT deployments we allow the aggregation node to be honest-but-curious or malicious and to collude with external parties.

Type I Attacker (A1): A malicious third party attempts to impersonate a legitimate user by forging secret values and replacing public keys; however, it neither knows the user’s partial private key nor the system’s master private key.

Type II Attacker (A2): A malicious Key Generation Center (KGC) attempts to impersonate a legitimate user; although it knows the system’s master private key and the user’s partial private key, it is unaware of the user’s secret values and public key.

Type III Attacker (A3): A colluding adversary that targets the aggregation procedure. A3 may control the aggregation node (AN) and collude with other entities. It can choose the aggregation set and order, deviate from the prescribed aggregation algorithm, and attempt to output an aggregate signature that verifies while containing forged or invalid components. However, A3 does not learn honest devices’ complete private keys unless it obtains them through the allowed oracle queries. The goal of A3 is to forge a valid aggregate signature that passes the verification equation.

Table 1 compares the attack characteristics of the three types of attackers. Additionally, the following oracles may be involved in the attackers’ attack processes:

1. Partial Private Key Generation Oracle (Ora-psk): The challenger C generates the partial private key of the user requested by the adversary A by executing the Partial-secret-key-generate algorithm.
2. Secret Value Generation Oracle (Ora-x): If the public key of the user has not been replaced, the challenger C may generate the secret value for the adversary A by executing the Secret-value-generate algorithm. However, if the public key of the user has been replaced, it returns (no result) directly.
3. Public Key Generation Oracle (Ora-pk): When the adversary A requests the public key of a user, the challenger C generates the public key of the user by executing the Public-key-generate algorithm.
4. Public Key Replacement Oracle (Ora-change): Subsequently, the adversary A replaces the public key of the target user with (the new public key, the original text does not specify the new public key, so it is retained as is); note that the Ora-x oracle must not be invoked again for the challenged user.
5. Signature Query Oracle (Ora-sign): When the adversary A requests the signature of a specific message from a user, the challenger C generates the signature by executing the Sign algorithm and sends the signature to the adversary A.

The adversary is allowed to query the above oracles adaptively, and may combine capabilities across different attack surfaces (e.g., public-key replacement together with chosen-message queries, or attempting to aggregate a mixture of valid and invalid signatures).

## 4. The Proposed Scheme

This section details the proposed lattice-based conditional privacy-preserving certificateless aggregate signature scheme. The symbols used in the scheme and their descriptions are shown in Table 2.

### 4.1. System Initialization

The system security parameter is defined as k. Additionally, the following parameters are required: a prime number q, positive integers $m,n,k,\theta \in R_{n,q}$. The aggregation node AN generates the corresponding public key $Apk\in Z_{q}^{n\times m}$ and private key $Ask\in Z_{q}^{m\times n}$ through a trapdoor function, satisfying $Apk\cdot Ask=qI_{n}\left(mod\;2q\right)$. The KGC selects $Mpk\in Z_{2q}^{m\times n}$ as the system master public key and generates the corresponding system master private key $Msk\in Z_{2q}^{n\times m}$ via a trapdoor function. Mpk and Msk satisfy $Mpk\cdot Msk=qI_{m}\left(mod\;2q\right)$. The KGC publicly releases the system master public key Mpk and keeps the system master private key Msk secret. Then, the KGC selects two hash functions for system computation: $H_{1}:{\left\{0,1\right\}}^{*}⟶B_{\omega }^{m}$ (a set of binary vectors with length m and weight ω), $H_{2}:{\left\{0,1\right\}}^{*}⟶D_{n}^{32}$ (all polynomials with the highest degree of n−1, where the vast majority of the coefficients of these polynomials are zero, with at most 32 non-zero coefficients, and the values of these non-zero coefficients are limited to ±1). The system public parameter is $Mparams=\left\{q,m,n,k,{H_{1},H}_{2}\right\}$.

### 4.2. Pseudonym Generation

The IIoT device sends its real identity identifier ${ID}_{i}$ to the KGC. The KGC records the validity period of the pseudonym as $T_{i1}$ and selects a random number $\alpha \in D_{\sigma }^{n}$ ($D_{\sigma }^{n}$ denotes a discrete Gaussian distribution with standard deviation σ) to calculate the signer’s pseudonym ${ANS}_{i}$:

(2) $${ANS}_{i}=H_{1}\left(Mpk\cdot \alpha \;mod\;2q,{ID}_{i}∥T_{i1}\right)$$

The KGC temporarily stores the device pseudonym information and stores the tuple (${ANS}_{i},\alpha ,T_{i1}$).

### 4.3. Partial Private Key Generation

The KGC randomly selects $b\in \left\{0,1\right\}$ and calculates the device’s partial private key:

(3) $$z_{i}=\alpha +{\left(-1\right)}^{b}Msk\cdot {ANS}_{i}$$

Finally, the KGC outputs $\left(z_{i},{ANS}_{i}\right)$ with a probability of $1/\left(M\;exp\left(-\frac{{∥Msk\cdot {ANS}_{i}∥}^{2}}{2\sigma^{2}}\right)cosh\left(\frac{z,Msk\cdot {ANS}_{i}}{\sigma^{2}}\right)\right)$, where M is the expected number of rejection-sampling iterations to ensure that ${ANS}_{i}$ is statistically independent of the master secret and $z_{i}$ is the partial private key of the pseudonym identity identifier ${ANS}_{i}$ for the IIoT smart device (M is the expected number of iterations of the partial private key generation algorithm). The KGC sends $\left\{{ANS}_{i},z_{i},T_{i1}\right\}$ to the IIoT smart device ${ID}_{i}$ and stores the tuple $\left({ANS}_{i},{z_{i},ID}_{i},\alpha ,T_{i1}\right)$.

### 4.4. Generation of Complete Public–Private Key Pair

When the IIoT smart device ${ID}_{i}$ receives $\left\{{ANS}_{i},z_{i},T_{i1}\right\}$, it first verifies the validity of the partial private key:

(4) $${ANS}_{i}=H_{1}\left(Mpk\cdot z_{i}+q{ANS}_{i}\;mod\;2q,{{ID}_{i}∥T}_{i1}\right)$$

If the verification succeeds, $\left\{{ANS}_{i},z_{i}\right\}$ is accepted; otherwise, it is rejected.

The IIoT smart device ${ID}_{i}$ selects a random value $u_{i}\in R_{n,1}$ as its own secret value and keeps it strictly confidential. The complete private key of the IIoT smart device ${ID}_{i}$ is ${SK}_{i}=\left(z_{i},u_{i}\right)$.

Finally, the public key of the IIoT smart device ${ID}_{i}$ is calculated:

(5) $${PK}_{i}=\theta \cdot z_{i}+u_{i}$$

The public–private key pair of ${ID}_{i}$ is $\left({SK}_{i},{PK}_{i}\right)$.

### 4.5. Signature Generation

When device ${ID}_{i}$ signs the message ${msg}_{i}$, it uses the public–private key pair $\left({SK}_{i},{PK}_{i}\right)$ corresponding to the pseudonym ${ANS}_{i}$. To generate the signature, it first selects $\left(y_{i1},y_{i2}\right)\in R_{n,k}$; then obtains the current timestamp $t_{1}$ and perform calculations:

(6) $$\Phi_{i}={\theta \cdot y}_{i1}+y_{i2}$$

(7) $$Q_{i}=H_{2}\left(\Phi_{i}{,{msg}_{i}∥t}_{i1}\right)$$

(8) $$s_{i1}=z_{i}\cdot Q_{i}+y_{i1}$$

(9) $$s_{i2}=u_{i}\cdot Q_{i}+y_{i2}$$

The timestamp $t_{i1}$ is used for freshness. The verifier accepts a signature if $t_{i1}$ falls within a tolerance window ΔT of its local time, which tolerates normal clock drift in distributed IIoT deployments. The IIoT smart device ${ID}_{i}$ first verifies whether $s_{i1}\;and\;s_{i2}$ belong to $R_{n,k-32}$. If the verification passes, the signature ${Sig}_{i}=\left(\Phi_{i},{Q_{i},s}_{i1},s_{i2}\right)$ for the message ${msg}_{i}$ is output. Then, the signer sends $M_{i}=\left\{{ANS}_{i},\theta ,{PK}_{i},{msg}_{i},{Sig}_{i},t_{i1}\right\}$ to the aggregation node.

### 4.6. Aggregate Signature Phase

When the aggregation node AN receives $\left\{M_{1},M_{2},\ldots ,M_{m}\right\}$, the aggregation node AN performs calculations:

(10) $$\Phi_{A}=\sum_{i=1}^{m}\Phi_{i}$$

Select a random number $\beta \in D_{\sigma }^{m}$, obtain the current timestamp $t_{A}$ and calculate:

(11) $$G_{A}=H_{1}\left(Apk\cdot \beta \;mod2q,\Phi_{A}∥t_{A}\right)$$

Here $G_{A}$ is the aggregate challenge derived from AN’s randomness and $\Phi_{A}$, binding the aggregate signature to the set of signatures included.

Randomly select $b\in \left\{0,1\right\}$ and calculate:

(12) $$S_{A}=\beta +{\left(-1\right)}^{b}Ask\cdot G_{A}$$

The aggregate signature ${Sig}_{A}=\left\{G_{A},S_{A},t_{A}\right\}$.

To reduce the amount of data transmission, AN calculates:

(13) $${S1}_{A}=\sum_{i=1}^{m}s_{i1}$$

(14) $${S2}_{A}=\sum_{i=1}^{m}s_{i2}$$

Finally, AN sends $\left\{S_{A},{G_{A},t_{A},{ANS}_{i},\theta ,Q}_{i},{PK}_{i},{S1}_{A},{S2}_{A},{msg}_{i}\right\}$ to the cloud verification platform.

### 4.7. Aggregate Signature Verification Phase

Upon receiving $\left\{S_{A},{G_{A},t_{A},{ANS}_{i},\theta ,Q}_{i},{PK}_{i},{S1}_{A},{S2}_{A},{msg}_{i}\right\}$, the cloud verification platform first calculates:

(15) $${QPK}_{A}=\sum_{i=1}^{m}Q_{i}\cdot {PK}_{i}$$

(16) $$B_{A}=\theta \cdot {S1}_{A}+{S2}_{A}-{QPK}_{A}$$

Then, the validity of the signature is verified using the following formula:

(17) $$G_{A}=H_{1}\left(Apk\cdot S_{A}+q\cdot G_{A}mod2q,B_{A}∥t_{A}\right)$$

If Equations (5)–(17) hold, then $\left\{{Sig}_{1},{Sig}_{2},\ldots ,{Sig}_{m}\right\}$ is a valid signature.

## 5. Security Analysis

### 5.1. Provable Security

Aggregated signatures are formed by combining multiple signatures and encrypting them using the private key of the aggregation node. To tamper with an aggregated signature, an attacker must first successfully forge an individual signature of the IISD. Based on the three attacker classes defined in Section 3.2., this section proves the security of the signature scheme by demonstrating its resistance to all three types of attackers under the random oracle model.

**Theorem** **1.** *Under the random oracle model, if an adversary A1 of Type I can successfully forge signatures within polynomial time to break the scheme’s unforgeability property, then there must exist another polynomial-time algorithm C. This algorithm can leverage adversary A1’s attack capabilities to effectively solve the DCK problem with a non-negligible advantage.*

**Proof.**  Assume an adversary A1 of Class I can invoke a polynomial-time algorithm, i.e., a challenger C, to forge signatures with non-negligible probability. The interaction between challenger C and adversary A1 proceeds as follows:

1. System Initialization. Challenger C runs the Setup algorithm, inputs security parameter k, outputs the system master private key Mpk and system master public key Mpk, and publishes Mpk and the system parameters Mparams. C maintains the following lists $L_{1},L_{2}$ for queries to hash functions $H_{1},H_{2}$; $L_{z}$ for partial private key queries; $L_{u}$ for secret value queries; $L_{PK}$ for user public key queries; $L_{SK}$ for signature key queries; and $L_{Sig}$ for signature queries. All the above lists are initially empty.
2. Queries. During the query phase, the attacker can perform corresponding query operations. Before the forgery phase, the identity ${ID}_{i}$ randomly selected by the attacker cannot be captured by the challenger.
  (a) $H_{1}$ Query. Challenger C creates a query list $L_{1}$, containing $\left\{{ANS}_{i},{ID}_{i},T_{i1}\right\}$. When attacker A1 requests anonymous identity information about ID, challenger C queries the list $L_{1}$. If it already exists, the challenge is aborted; if not, the corresponding pseudonym ${ANS}_{i}$ is calculated and generated as a response, and stored in the list $L_{1}$
  (b) $H_{2}$ Query. Challenger C creates a query list $L_{2}$, containing $\left\{msg,t_{i1},Q_{i}\right\}$. When attacker A1 requests a signature for the message msg, challenger C queries the list $L_{2}$. If the $Q_{i}$ already exists, the value is directly returned to A1; otherwise, the attacker calculates the $Q_{i}$ value, returns it to A1, and adds it to the list $L_{2}$
  (c) Partial private key query. The challenger C creates a query list $L_{z}$, which contains $\left\{{ANS}_{i},z_{i}\right\}$. Attacker A1 obtains the partial private key with the identity ${ANS}_{i}$ by sending a query to challenger C. After receiving the query request, challenger C searches the list $L_{z}$. If it exists, $z_{i}$ is returned to A1; if not, challenger C calculates and generates $z_{i}$ for A1 based on the anonymous identity information ${ANS}_{i}$ and stores it in the list $L_{z}$
  (d) Secret value query. The challenger C creates a query list $L_{u}$, which contains $\left\{{ANS}_{i},u_{i}\right\}$. Attacker A1 obtains the secret value with the identity ${ANS}_{i}$ by sending a query to challenger C. After receiving the query request, challenger C searches the list $L_{u}$. If it exists, $u_{i}$ is returned to A1; if not, challenger C randomly selects $u_{i}$ for A1 based on the anonymous identity information ${ANS}_{i}$ and stores it in the list $L_{u}$
  (e) Public key query. The challenger C creates a query list $L_{pk}$, which contains $\left\{{ANS}_{i},{PK}_{i}\right\}$. Attacker A1 obtains the public key with the identity ${ANS}_{i}$ by sending a query to challenger C. After receiving the query request, challenger C searches the list $L_{PK}$. If it exists, ${PK}_{i}$ is returned to A1; if not, challenger C calculates the user’s public key ${PK}_{i}=\theta \cdot z_{i}+u_{i}$ based on the partial private key and the secret value, and stores it in the list $L_{PK}$
  (f) Public key replacement query. The attacker selects a new public key ${PK}_{i}^{*}$ for the identity ${ANS}_{i}$ to replace the original public key. When challenger C receives the public key replacement query from attacker A1 for the identity ${ANS}_{i}$, $L_{PK}$ is updated to $\left({ANS}_{i},{PK}_{i}^{*}\right)$
3. Forgery. Attacker A1 claims, with a non-negligible probability, to be of identity ${ANS}_{i}$ and public key ${PK}_{i}^{*}$, and outputs a successfully forged signature ${Sig}^{*}=\left(Q_{i},s_{i1}^{*},s_{i2}^{*}\right)$ on message ${msg}_{i}$. When an adversary can successfully forge a new signature, according to the Forking Lemma [23], the challenger can output another valid signature ${Sig}^{\prime }=\left(Q_{i},s_{i1}^{\prime },s_{i2}^{\prime }\right)$ on message ${msg}_{i}$ with identity ${ANS}_{i}$ with a non-negligible probability.

The above two signatures satisfy:

(18) $$\theta \cdot s_{i1}^{*}+s_{i2}^{*}-Q_{i}^{*}\cdot {PK}_{i}^{*}=\theta \cdot s_{i1}^{\prime }+s_{i2}^{\prime }-Q_{i}^{\prime }\cdot {PK}_{i}^{*}$$

It can be deduced from (5):

(19) $$\theta \cdot s_{i1}^{*}+s_{i2}^{*}-Q_{i}^{*}\cdot \left(\theta \cdot z_{i}^{*}+u_{i}^{*}\right)=\theta \cdot s_{i1}^{\prime }+s_{i2}^{\prime }-Q_{i}^{\prime }\cdot \left(\theta \cdot z_{i1}^{*}+u_{i1}^{*}\right)\;⟹\theta \left(s_{i1}^{*}-s_{i1}^{\prime }+Q_{i}^{\prime }z_{i1}^{*}-Q_{i}^{*}z_{i1}^{*}\right)+\left(s_{i2}^{*}-s_{i2}^{\prime }+Q_{i}^{\prime }u_{i}^{*}-Q_{i}^{*}u_{i}^{*}\right)=0$$

Since the coefficients of $Q,z_{id},s_{i},u_{id}$ are all very small, we find two polynomials $f_{1}=\left(s_{i1}^{*}-s_{i1}^{\prime }+Q_{i}^{\prime }z_{i}^{*}-Q^{*}z_{i}^{*}\right)$, $f_{2}=\left(s_{i2}^{*}-s_{i2}^{\prime }+Q_{i}^{\prime }u_{i}^{*}-Q^{*}u_{i}^{*}\right)$ such that $\theta \cdot f_{1}+f_{2}=0$. Through [24], lemma 3.7, we know that such polynomials allow us to solve the DCK problem. Therefore, under the random oracle model and the DCK problem assumption, through the above security treaty proof, it can be seen that when facing the adaptive chosen-message attack, chosen-identity attack, and public key replacement attack implemented by the first-type attacker A1, this scheme meets the unforgeability security requirements. □

**Theorem** **2.** *Under the random oracle model, if a Type II adversary A2 exists that can successfully forge signatures in probabilistic polynomial time, thereby compromising the non-forgeability property of this scheme, then there must exist another probabilistic polynomial-time algorithm C. This algorithm can effectively solve the DCK problem with a non-negligible advantage by exploiting the attack capabilities of adversary A2.*

**Proof.**  Assume that a Type II adversary A2 can invoke a polynomial-time algorithm, i.e., a challenger C, to forge signatures with non-negligible probability. The interaction between challenger C and adversary A2 proceeds as follows:

1. System initialization. The challenger C runs the Setup algorithm, inputs the security parameter k, and outputs the system master private key Msk and the system master public key Mpk. Then, C publishes Mpk and the system parameters Mparams. C maintains the following lists $L_{1},L_{2}$ for queries to hash functions $H_{1},H_{2}$; $L_{z}$ for partial private key queries; $L_{u}$ for secret value queries; $L_{PK}$ for user public key queries; $L_{SK}$ for signature key queries; and $L_{Sig}$ for signature queries. All the above lists are initially empty.
2. Queries. In the query phase, the attacker can perform corresponding query operations. Before the forgery phase, the identity ${ID}_{i}$ randomly selected by the attacker cannot be captured by the challenger.
  (a) $H_{1}$ query. The challenger C creates a query list $L_{1}$, which contains $\left\{{ANS}_{i},{ID}_{i},T_{1i}\right\}$. When attacker A2 requests an anonymous identity information query about ID, challenger C checks the list $L_{1}$. If it already exists, the challenge is aborted; if not, the corresponding pseudonym ${ANS}_{i}$ is calculated and generated as a response, and stored in the list $L_{1}$
  (b) $H_{2}$ query. The challenger C creates a query list $L_{2}$, which contains $\left\{msg,t_{i1},Q_{i}\right\}$. When attacker A2 requests a signature for message msg, challenger C checks the list $L_{2}$. If Q already exists, the value is directly returned to A2; otherwise, the attacker calculates the Q value, returns it to A2, and adds it to the list $L_{2}$
  (c) Partial private key query. The challenger C creates a query list $L_{z}$, which contains $\left\{{ANS}_{i},z_{i}\right\}$. Attacker A2 obtains the partial private key with the identity ${ANS}_{i}$ by sending a query to challenger C. After receiving the query request, challenger C searches the list $L_{z}$. If it exists, *z_i_* is returned to A2; if not, challenger C calculates and generates $z_{i}$ for A2 based on the anonymous identity information ${ANS}_{i}$ and stores it in the list $L_{z}$
  (d) Secret value query. The challenger C creates a query list $L_{u}$, which contains $\left\{{ANS}_{i},u_{i}\right\}$. Attacker A2 obtains the secret value with the identity ${ANS}_{i}$ by sending a query to challenger C. After receiving the query request, challenger C searches the list $L_{u}$. If it exists, $u_{id}$ is returned to A2; if not, challenger C randomly selects $u_{i}$ for A2 based on the anonymous identity information ${ANS}_{i}$ and stores it in the list $L_{u}$
  (e) Public key query. The challenger C creates a query list $L_{pk}$, which contains $\left\{{ANS}_{i},{PK}_{i}\right\}$. Attacker A1 obtains the public key with the identity ${ANS}_{i}$ by sending a query to challenger C. After receiving the query request, challenger C searches the list $L_{PK}$. If it exists, ${PK}_{i}$ is returned to A1; if not, challenger C calculates the user’s public key ${PK}_{i}=\theta \cdot z_{i}+u_{i}$ based on the partial private key and the secret value, and stores it in the list $L_{PK}$
3. Signature forgery. Given a message ${msg}_{i}$, the challenger C first browses $L_{u}$ to find the user’s private key ${SK}_{i}^{*}$, and outputs a successfully forged signature ${Sig}^{*}=\left(Q_{i},s_{i1}^{*},s_{i2}^{*}\right)$ for the message ${msg}_{i}$. When an adversary can successfully forge a new signature, according to the Forking Lemma, the challenger can output another valid signature ${Sig}^{\prime }=\left(Q_{i},s_{i1}^{\prime },s_{i2}^{\prime }\right)$ on the message ${msg}_{i}$ with identity ${ANS}_{i}$ with a non-negligible probability. The above two signatures satisfy:

(20) $$\theta \cdot s_{i1}^{*}+s_{i2}^{*}-Q_{i}^{*}\cdot {PK}_{i}^{*}=\theta \cdot s_{i1}^{\prime }+s_{i2}^{\prime }-Q_{i}^{\prime }\cdot {PK}_{i}^{*}$$

It can be deduced from (5):

(21) $$\theta \cdot s_{i1}^{*}+s_{i2}^{*}-Q_{i}^{*}\cdot \left(\theta \cdot z_{i}^{*}+u_{i}^{*}\right)=\theta \cdot s_{i1}^{\prime }+s_{i2}^{\prime }-Q_{i}^{\prime }\cdot \left(\theta \cdot z_{i}^{*}+u_{i}^{*}\right)\;⟹\theta \left(s_{i1}^{*}-s_{i1}^{\prime }+Q_{i}^{\prime }z_{i1}^{*}-Q_{i}^{*}z_{i1}^{*}\right)+\left(s_{i2}^{*}-s_{i2}^{\prime }+Q_{i}^{\prime }u_{i}^{*}-Q_{i}^{*}u_{i}^{*}\right)$$

Since the coefficients of $Q,z_{i},s_{in},u_{i}$ are all very small, we find two polynomials $f_{1}=\left(s_{i1}^{*}-s_{i1}^{\prime }+Q_{i}^{\prime }z_{i}^{*}-Q_{i}^{*}z_{i}\right)$, $f_{2}=\left(s_{i2}^{*}-s_{i2}^{\prime }+Q_{i}^{\prime }u_{i}^{*}-Q_{i}^{*}u_{i}^{*}\right)$ such that $\theta \cdot f_{1}+f_{2}=0$. Through [24], lemma 3.7, we know that such polynomials enable us to solve the DCK problem. Therefore, under the random oracle model and the DCK problem assumption, the security protocol proof demonstrates that this scheme satisfies the unforgeability security requirement against adaptive selective message attacks and selective identity attacks carried out by a second-class attacker A2. □

Based on the two theorems above, an adversary cannot successfully forge any individual signature and therefore cannot construct a valid aggregate signature.

**Theorem** **3.** *Under the random oracle model, if an adversary A3 of Type III exists that can successfully forge aggregate signatures in probabilistic polynomial time, thereby compromising the unforgeability property of this scheme, then there necessarily exists a probabilistic polynomial-time algorithm C. This algorithm can effectively solve the RSIS problem with a non-negligible advantage by exploiting the attack capabilities of adversary A3.*

**Proof.**  Assume that a Type III adversary A3 can invoke a polynomial-time algorithm, i.e., challenger C, to forge aggregate signatures with non-negligible probability. The interaction between challenger C and adversary A3 proceeds as follows:

1. System Initialization. The challenger C runs the Setup algorithm, inputs the security parameter k, and outputs the system master private key Msk and the system master public key Mpk. Then, C publishes Mpk and the system parameters Mparams. C maintains the following lists: $L_{1},L_{2}$ for queries to hash functions $H_{1},H_{2}$; $L_{z}$ for partial private key queries; $L_{u}$ for secret value queries; $L_{PK}$ for user public key queries; $L_{SK}$ for signature key queries; and $L_{Sig}$ for signature queries. All the above lists are initially empty.
2. Queries. In the query phase, the attacker can perform corresponding query operations. Before the forgery phase, the identity ${ID}_{i}$ randomly selected by the attacker cannot be captured by the challenger.
  (a) $H_{1}$ Query. The challenger C creates a query list $L_{1}$, which contains $\left\{{ANS}_{i},{ID}_{i},T_{i1}\right\}$. When attacker A3 requests an anonymous identity information query about ID, challenger C checks the list $L_{1}$. If it already exists, the challenge is aborted; if not, the corresponding pseudonym ${ANS}_{i}$ is computed and generated as a response, and stored in the list $L_{1}$
  (b) $H_{2}$ Query. The challenger C creates a query list $L_{2}$, which contains $\left\{msg,t_{i1},Q_{i}\right\}$. When attacker A3 requests a signature for message msg, challenger C checks the list $L_{2}$. If $Q_{i}$ already exists, the value is directly returned to A3; otherwise, the attacker computes the $Q_{i}$ value, returns it to A3, and adds it to the list $L_{2}$
  (c) Partial Private Key Query. The challenger C creates a query list $L_{z}$, which contains $\left\{{ANS}_{i},z_{i}\right\}$. Attacker A3 obtains the partial private key with identity ${ANS}_{i}$ by sending a query to challenger C. After receiving the query request, challenger C searches the list $L_{z}$. If it exists, *z_i_* is returned to A3; if not, challenger C computes and generates $z_{i}$ for A3 based on the anonymous identity information ${ANS}_{i}$ and stores it in the list $L_{z}$
  (d) Secret Value Query. The challenger C creates a query list $L_{u}$, which contains $\left\{{ANS}_{i},u_{i}\right\}$. Attacker A3 obtains the secret value with identity ${ANS}_{i}$ by sending a query to challenger C. After receiving the query request, challenger C searches the list $L_{u}$. If it exists, $u_{i}$ is returned to A3; if not, challenger C randomly selects $u_{i}$ for A3 based on the anonymous identity information ${ANS}_{i}$ and stores it in the list $L_{u}$
  (e) Public Key Query. The challenger C creates a query list $L_{pk}$, which contains $\left\{{ANS}_{i},{PK}_{i}\right\}$. Attacker A3 obtains the public key with identity ${ANS}_{i}$ by sending a query to challenger C. After receiving the query request, challenger C searches the list $L_{PK}$. If it exists, ${PK}_{i}$ is returned to A3; if not, challenger C calculates the user’s public key ${PK}_{i}=\theta \cdot z_{i}+u_{i}$ based on the partial private key and the secret value, and stores it in the list $L_{PK}$
  (f) Signature Query: When challenger C receives a signature query from attacker A3 about identity ${ANS}_{i}$, if $\left({ANS}_{i},msg,{Sig}_{i}\right)$ exists in $L_{Sig}$, ${Sig}_{i}$ is returned to the attacker; otherwise, challenger C randomly selects $\left(y_{i1},y_{i2}\right)\in R_{n,k}$, calculates the signature ${Sig}_{i}=\left(\Phi_{i},s_{i1},s_{i2}\right)$, inserts the tuple $\left({ANS}_{i},msg,{Sig}_{i}\right)$ into the list $L_{Sig}$, and returns ${Sig}_{i}$
3. Forgery. Through the above query process, under the same state information, A3 obtains the signatures $\left\{{Sig}_{1},{Sig}_{2},\ldots ,{Sig}_{n}\right\}$ of *n* users with identity information $\left\{{ID}_{1},{ID}_{2},\ldots ,{ID}_{n}\right\}$ and corresponding public keys $\left\{{PK}_{1},{PK}_{2},\ldots ,{PK}_{n}\right\}$ on different messages $\left\{{msg}_{1},{msg}_{2},\ldots ,{msg}_{n}\right\}$. Next, attacker A3 attempts to forge a valid aggregate signature $\left\{S_{A}^{*},G_{A}^{*},t_{A}\right\}$. When the adversary can successfully forge a new signature, according to the Forking Lemma, the challenger can output another valid aggregate signature $\left\{S_{A}^{\prime },G_{A}^{\prime },t_{A}\right\}$ with a non-negligible probability.

According to the aggregate signature verification formula:

(22) $$Apk\cdot S_{A}^{*}+qG_{A}^{*}\equiv Apk\cdot S_{A}^{\prime }+qG_{A}^{\prime }\;\left(mod\;2q\right)$$

It can be transformed into:

(23) $$Apk\cdot \left(S_{A}^{*}-S_{A}^{\prime }\right)+q\left(G_{A}^{*}-G_{A}^{\prime }\right)\equiv 0\;\left(mod\;2q\right)$$

Since $G_{A}^{*}\neq G_{A}^{\prime }$ and q is a prime number, there exists an inverse $q\cdot q^{-1}\equiv 1\;mod2$, and we can solve:

(24) $$Apk\cdot \left(S_{A}^{*}-S_{A}^{\prime }\right)\cdot q^{-1}=-\left(G_{A}^{*}-G_{A}^{\prime }\right)\;mod\;2$$

If $\left(G_{A}^{*}-G_{A}^{\prime }\right)$ belongs to the null space of Apk (i.e., there exists a vector y such that $Apk\cdot y\equiv \left(G_{A}^{*}-G_{A}^{\prime }\right)\;mod\;q$, then we can construct:

(25) $$x=\left(S_{A}^{*}-S_{A}^{\prime }\right)\cdot q^{-1}+y$$

Then:

(26) $$Apk\cdot x=Apk\cdot \left[\left(S_{A}^{*}-S_{A}^{\prime }\right)\cdot q^{-1}+y\right]=-\left(G_{A}^{*}-G_{A}^{\prime }\right)+\left(G_{A}^{*}-G_{A}^{\prime }\right)=0$$

Therefore, x is an instance of a solution to the RSIS problem. Under the random oracle model and the RSIS problem assumption, through the above security treaty proof, it can be seen that when facing the adaptive chosen-message attack and chosen-identity attack implemented by the third-type attacker A3, this aggregate signature scheme meets the unforgeability security requirements. □

A combined attacker may try to mix strategies, such as forging a missing individual signature while simultaneously outputting an aggregate over the whole set or crafting an aggregate that “hides” invalid components. However, any successful combined attack must eventually manifest as either (i) a forgery of at least one new individual signature (covered by Theorems 1 and 2 under DCK), or (ii) a forgery of a valid aggregate signature that passes Equation (17) without all valid constituents (covered by Theorem 3 under RSIS). Therefore, the three security reductions jointly rule out combined or adaptive forgery strategies in the random oracle model.

### 5.2. Security Performance Analysis

#### 5.2.1. Non-Repudiation

In this scheme, messages are signed using the private key ${SK}_{i}$ of the IISD, and only the corresponding public key ${PK}_{i}$ can validate the signature’s validity. Since the private key is held exclusively by the signer, once the signature is verified, it is conclusively established that the signer signed the message, making repudiation impossible. Additionally, the scheme incorporates a timestamp to record the signing time, preventing the signer from later repudiating the signature by claiming time discrepancies.

#### 5.2.2. Conditional Privacy Protection

In this scheme, KGC creates pseudonymous identity identifiers ${ANS}_{i}$ for IISDs. No entity other than KGC can recover the device’s true identity information. This prevents industrial devices from having their location monitored during certain activities, making it impossible or extremely difficult for external attackers to link specific behaviors, events, or data to a particular device.

If a malicious IISD sends false or malicious messages, the KGC can identify the IISD’s true device ID by consulting its stored list $\left({ANS}_{i},{ID}_{i},\alpha ,T_{i1}\right)$, thereby enabling conditional traceability and accountability.

#### 5.2.3. Unlinkability

Unlinkability further requires that an adversary cannot decide whether two valid signatures were produced by the same device, even if the device identity remains hidden. LCP-CAS achieves unlinkability across validity periods by rotating pseudonyms: for each period $T_{i1}$, the device uses a fresh pseudonym ${ANS}_{i}$ and the corresponding key pair to generate signatures, so signatures from different periods are not linkable via public information. Thus, attackers cannot determine whether multiple intercepted messages originate from the same IISD.

#### 5.2.4. Forward Security

In this scheme, the validity of pseudonymous identities ${ANS}_{i}$ is time-bound. The IISD’s public–private key pair is generated using anonymous identity information, achieving time-bound key validity. This prevents future keys from decrypting past messages.

#### 5.2.5. Impersonation Attacks

To impersonate a legitimate signer, an attacker must generate a valid forged signature. Based on Theorems 1–3, such forgery is impossible. Thus, this scheme resists impersonation attacks.

#### 5.2.6. Tampering Attacks

This scheme utilizes a hash function in the signature generation process. When data integrity verification is required, the hash value of the data is recalculated and compared with the previously generated hash value. If the two hash values are inconsistent, it indicates that the message has been tampered with.

#### 5.2.7. Replay Attacks

In this scheme, timestamps are used both in the interaction between the KGC (Key Generation Center) and the signer, and in the interaction between the signer and the verifier. Upon receiving the data, the system can verify whether the timestamp falls within a reasonable range. If the timestamp of the data shows that the data was sent a long time ago, the system can determine this as a replay attack and reject the message.

#### 5.2.8. Prevention of Master Private Key Statistical Attacks

In this scheme, a rejection sampling mechanism is introduced when outputting the user’s partial private key $z_{i}$. Malicious users cannot infer the statistical characteristics of the system master private key Msk by analyzing the partial private keys $z_{i}$ of multiple users. In addition, rejection sampling ensures that the distribution of $z_{i}$ is indistinguishable from that of a random sample, thereby avoiding the leakage of master private key information.

#### 5.2.9. Resistance to Collusion Attacks

The partial private key $z_{i}$ of each device is generated by the KGC through a random number α and is bound to the device’s pseudonym ${ANS}_{i}$. The complete private key ${SK}_{i}=\left(z_{i},u_{i}\right)$ contains a random value $u_{i}$, which is generated locally by the device and strictly kept secret. Since α is generated randomly, there is no direct correlation between the partial private keys of different devices. Even if an attacker obtains the partial private keys of multiple devices, they cannot derive the master private key Msk of the KGC or the private keys of other devices. The independence of private keys makes it impossible for collusion attackers to derive the private keys of other devices by sharing private key information.

## 6. Performance Analysis

This section compares the proposed scheme with existing lattice-based aggregate signature schemes in terms of security properties, communication costs, and computational overhead.

### 6.1. Security Feature Comparison

Table 3 provides a detailed comparative analysis of security features between the proposed solution and several existing lattice-based aggregate signature schemes.

The existing schemes exhibit distinct performance stratification in terms of security. Although the schemes in References [25,26] possess security attributes such as post-quantum security and non-repudiation, they still have several security flaws. The schemes in References [27,28] have obvious shortcomings in multiple security dimensions. By comparison, the proposed scheme in this paper not only remedies all known security vulnerabilities but also enhances the security protection level through an innovative defense mechanism, achieving an overall improvement in security performance.

From the specific comparison, in terms of privacy protection, the proposed scheme realizes both conditional privacy protection and unlinkability by means of dynamic pseudonym binding and timestamp technology, overcoming the defects in unlinkability of the schemes in References [27,28]. In terms of dynamic security, it adopts a forward—secure key evolution mechanism and time—validity control of pseudonym identities, solving the deficiencies of the scheme in Reference [28] in forward security and replay attack resistance. In terms of anti-security attack capability, it innovatively applies the bimodal Gaussian rejection sampling technology, making it the only scheme that can effectively prevent statistical attacks. These innovations not only fill all the security loopholes of existing schemes but also elevate the overall security protection capability to a new level.

### 6.2. Computational Overhead Comparison

This part presents a systematic comparison of time overhead between the proposed scheme and existing schemes through a combination of theoretical analysis and experimental validation.

#### 6.2.1. Theoretical Analysis

First, based on the fundamental operation symbols defined in Table 4, we constructed a formal computational complexity analysis model. This model quantitatively compares the time complexity of each scheme during the two core phases—signature generation and verification—from a theoretical perspective.

According to the analysis results in Table 5, the proposed aggregate signature scheme exhibits computational-efficiency advantages, primarily reflected in reduced computational complexity during the aggregation and verification phases. The efficiency of LCP-CAS mainly stems from two design choices. First, unordered aggregation is realized without intersection computation, avoiding the high overhead typically incurred by intersection-based lattice aggregation. Second, the challenge $Q_{i}$ output by $H_{2}$ is a sparse polynomial (with at most 32 non-zero coefficients in {±1}), which makes products lightweight in practice. As a result, aggregation and verification are dominated by a small constant number of matrix-vector multiplications together with linear-time additions.

In the aggregation phase, the scheme requires only two matrix-vector multiplications, one hash operation, one vector addition, and polynomial-ring addition, making it more efficient than schemes that rely on Gaussian sampling, polynomial-ring multiplication, or modular arithmetic.

During verification, the scheme requires only 3 matrix multiplications, 1 hash operation, 2 vector additions, (n−1) polynomial ring addition, and 1 vector subtraction. Its low fixed cost and linear scaling, which relies solely on lightweight addition operations, substantially outperform alternatives (e.g., schemes in [27,28] that depend on high-overhead polynomial ring multiplication or modular arithmetic). Consequently, the proposed scheme delivers superior performance, higher computational efficiency, and stronger scalability in large-scale signature aggregation scenarios, making it well-suited for practical deployment.

#### 6.2.2. Experimental Analysis

**Figure 3:**
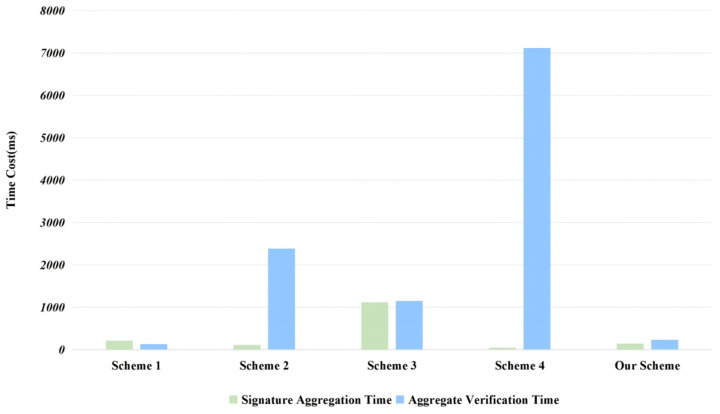
Time cost comparison of signature aggregation and aggregate verification among prior schemes and our scheme. The prior schemes correspond to Scheme 1 [25], Scheme 2 [26], Scheme 3 [27], Scheme 4 [28].

This experiment was conducted on a laptop equipped with 16 GB of memory and an Intel^®^ Core™ i5-13500H processor. We employed SageMath to write code simulating a key generation center for partial private key computation and distribution. Additionally, the signature and signature verification processes were simulated using the Rust programming language. Throughout this paper, the proposed scheme will continue to undergo computational overhead comparisons with the aforementioned four schemes, as illustrated in Figure 3. Regarding computational overhead, the primary metrics tracked are the time required to generate the aggregate signature and the time required to verify the aggregate signature.

To establish a unified benchmark for comparison, we normalize the runtimes reported in prior papers into estimated execution times on i5-13500H setup with (*n* = 512, *q* = 8,383,489). We first normalize CPU performance using the ratio of PassMark single-core scores. We then scale with respect to *n* according to the dominant operation in each stage: Stages dominated by matrix-vector operations, which have a time complexity of $O(n^{2})$, are scaled as $n^{2}$. In contrast, stages dominated by NTT-based polynomial multiplication, which have a time complexity of O(nlogn), are scaled as nlogn. Finally, we adjust for modulus-dependent arithmetic by scaling proportionally to the modulus bit-length $\left\lceil \log_{2}q\right\rceil$. Minor linear-time components such as hashing and vector additions are treated as negligible compared with the dominant lattice arithmetic. This yields a unified and reproducible basis for cross-scheme performance comparison.

In the performance evaluation of signature aggregation schemes, when aggregating 100 signatures, our scheme completes aggregation in 143 ms. While slower than the fastest approaches in [28] (46 ms) and [26] (113 ms), it is faster than [25] (214 ms) and [27] (1120 ms), with efficiency improvements of 33.2% and 87.2%, respectively. Regarding verification performance, our approach completes verification in 232 ms. While its verification efficiency is second only to the optimal approach in [25] (131 ms), it is 4.96 times faster than [27] (1150 ms), 10.3 times faster than [26] (2383 ms), and 30.7 times faster than [28] (7115 ms).

A detailed analysis of the performance characteristics reveals that although [28] achieves the fastest aggregate signature generation, its excessive verification time may cause severe performance bottlenecks in practical systems; [26] exhibits high signature efficiency but its verification time exceeds 10 times that of our approach; [25] delivers the fastest verification but its signature efficiency is significantly lower than ours. In contrast, the proposed scheme achieves the optimal balance between the two critical performance metrics of aggregation signing and verification.

**Figure 4:**
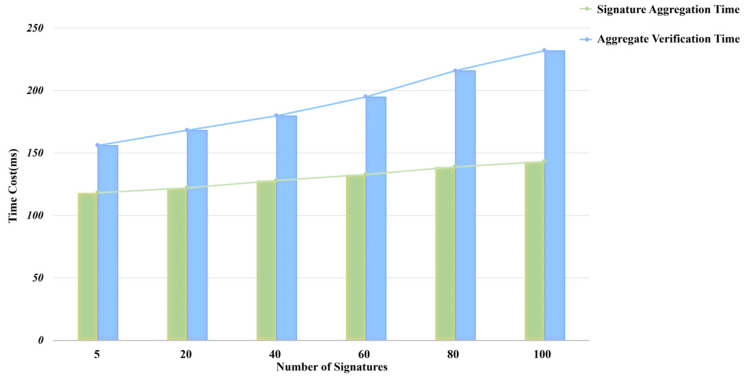
Time variation of LCP-CAS scheme.

To quantify performance at different scales, we experimentally measured processing times for signature counts ranging from 5 to 100. As shown in Figure 4, with increasing signature counts, the proposed scheme exhibits excellent linear growth characteristics in both aggregation signing and verification efficiency, fully demonstrating its scalability and stability. For aggregated signing, processing time increased from 118 ms to 143 ms—a modest 21.2% rise. For verification, time consumption grew from 156 ms to 232 ms, representing a 48.7% increase. This near-linear time growth curve indicates that our approach successfully avoids complexity explosion in algorithm design, achieving near-ideal O(n) time complexity.

**Figure 5:**
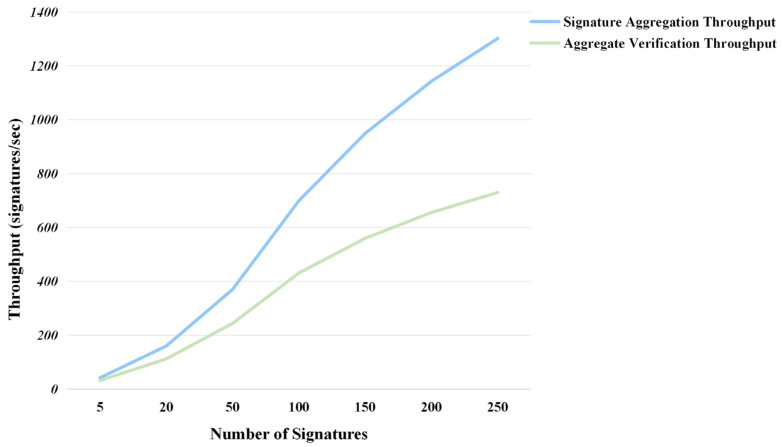
Throughput performance of LCP-CAS scheme.

To further evaluate the system performance in large-scale IIoT deployment scenarios, we introduce throughput as a critical performance metric, defined as the number of signatures processed per second. This metric provides a comprehensive assessment of the system’s processing capacity from a systemic perspective, effectively complementing traditional time consumption analysis. We extended the experimental scale to 250 signatures to comprehensively evaluate the scheme’s performance in bulk processing scenarios. The experimental results presented in Figure 5 demonstrate that the LCP-CAS scheme exhibits excellent throughput characteristics across different signature scales.

The extended experimental results reveal that the LCP-CAS scheme maintains excellent performance even in large-scale signature processing. As the number of signatures increases from 5 to 250, the generation throughput significantly improves from 42.4 signatures/s to 1302.1 signatures/s, representing approximately 30-fold growth, while the verification throughput increases from 32.1 signatures/s to 730.0 signatures/s, representing approximately 22-fold growth. This near-linear growth trend demonstrates the scheme’s excellent scalability.

Compared with similar solutions, it shows stable throughput across different signature scales. First, the time growth remains gradual, preventing performance degradation despite increased signatures. Second, both signing and verification efficiency consistently stay at high levels, meeting real-time requirements. Finally, system resource consumption growth is controllable, which is crucial for practical deployment.

## 7. Conclusions

This paper has proposed LCP-CAS, a lattice-based conditional privacy-preserving certificateless aggregate signature scheme tailored for Industrial Internet of Things (IIoT) environments. The scheme integrates a pseudonym-based mechanism to safeguard device identity privacy, incorporating timestamps and rejection sampling to mitigate replay and statistical attacks while preserving post-quantum security. Formal security analysis under the random oracle model demonstrates that LCP-CAS resists forgery attacks from three adversarial types and satisfies essential security properties including integrity, non-repudiation, conditional privacy preservation, and collusion resistance. Performance evaluation further confirms its scalability: aggregating and verifying 100 signatures requires only 143 ms and 232 ms, respectively, with gradual performance degradation, stable processing efficiency, and linearly increasing resource consumption as the number of signatures grows. These characteristics render LCP-CAS particularly suitable for large-scale IIoT deployments such as smart factories and equipment monitoring systems.

Despite these advantages, certain limitations merit further investigation. Conditional privacy in LCP-CAS is achieved via a pseudonym-identity mapping maintained by the KGC, thereby placing the mapping database and key-distribution channel within the system’s trust boundary. Moreover, the unordered aggregation process assumes reliable collection of signature components by the CVP, introducing potential availability risks in the presence of malicious or unstable aggregators. Practical deployment also necessitates careful parameter tuning, as the efficiency of rejection and discrete Gaussian sampling is influenced by hardware characteristics of resource-constrained devices. Future work will explore distributed or auditable KGC architectures to reduce reliance on a single trust entity, enhance robustness against malicious aggregators through verifiable inclusion proofs or multi-AN redundancy, and optimize implementation constants via efficient Gaussian samplers and polynomial arithmetic, followed by evaluation on representative IIoT hardware platforms.

## Figures and Tables

**Table 1:** Comparison of three types of attackers.

Characteristic	Type-I Adversary	Type-II Adversary	Type-III Adversary
Access to System Master Key	No	Yes	Yes
Replace User Public Keys	Yes	No	No
Compute User Partial Key	No	Yes	Yes
Attack Goal	Forge a Signature	Forge a Signature	Forge an Aggregate Signature

**Table 2:** Symbols and their descriptions.

Notation	Description
n	Polynomial degree
q	Prime number
σ	Standard deviation for discrete Gaussian samplers
α	KGC’s random Gaussian sample used in pseudonym/partial-key generation
*k*	Security parameter
Mparams	Public parameters
$R_{q}=Z_{q}\left[X\right]/X^{n}+1$	$\mathrm{Polynomial}\;\mathrm{ring}\;\mathrm{over}\;Z_{q}$
${ID}_{i}$	Device identity information
${ANS}_{i}$	Device pseudonym
${msg}_{i}$	Message to be signed
$T_{i1}$	Pseudonym validity period
Mpk	System master public key
Msk	System master private key
${PK}_{i}$	Device public key
${SK}_{i}$	Device private key
Apk	Aggregation node public key
Ask	Aggregation node private key
$\Phi_{i}$	Aggregate intermediate commitments
$z_{i}$	Device partial private key
$u_{i}$	Device secret value
${Sig}_{i}$	Individual signature
${Sig}_{A}$	Aggregate signature

**Table 3:** Comparison of safety characteristics of LCP-CAS scheme. √ denotes supported; × denotes not supported.

	Scheme [25]	Scheme [26]	Scheme [27]	Scheme [28]	Our Scheme
Non-Repudiation	√	√	√	√	√
Unlinkability	√	√	×	×	√
Conditional Privacy	√	√	×	×	√
No Key Escrow	√	√	×	√	√
Impersonation Attacks	√	√	√	√	√
Statistical Attacks	×	×	×	×	√
Replay Attacks	√	√	×	×	√
Tampering Attacks	√	√	√	√	√
Forward Security	√	×	×	×	√
Post-Quantum Security	√	√	√	√	√

**Table 4:** Symbol description.

Notation	Description
$T_{h}$	Hash operation
$T_{\mathrm{add}}$	Vector addition
$T_{\mathrm{sub}}$	Vector subtraction
$T_{\mathrm{sam}}$	Polynomial ring sampling
$T_{\mathrm{mi}}$	Matrix intersection computation
$T_{\mathrm{mul}}$	Matrix-vector multiplication
$T_{\mathrm{pm}}$	Polynomial ring multiplication
$T_{\mathrm{pa}}$	Polynomial ring addition
$T_{\mathrm{ps}}$	Polynomial ring subtraction
$T_{g}$	Gaussian sampling
$T_{\mathrm{mod}}$	Modular operation
$T_{\mathrm{bit}}$	High/Low-bit extraction

**Table 5:** Theoretical comparison of LCP-CAS scheme time overhead.

Scheme	Signature Aggregation	Aggregate Verification
Scheme [25]	$2\left(n-1\right)T_{\mathrm{add}}+T_{\mathrm{bit}}$	${2T}_{\mathrm{mul}}+2T_{h}+{2T}_{\mathrm{bit}}+T_{\mathrm{sub}}$
Scheme [26]	$\left(n-1\right)T_{\mathrm{add}}$	$\left(n+1\right)T_{h}+{\left(n+1\right)T}_{\mathrm{mul}}$
Scheme [27]	$2T_{\mathrm{sam}}+2T_{h}+2T_{\mathrm{pm}}+2T_{\mathrm{pa}}+2T_{\mathrm{mod}}$	$\left(n+2\right)T_{h}+\left(2n+2\right)T_{\mathrm{pm}}$ $+\left(n+1\right)T_{\mathrm{pa}}+{\left(n+1\right)T}_{\mathrm{ps}}$
Scheme [28]	$2T_{\mathrm{mi}}+2T_{g}+2T_{\mathrm{add}}$	${3\mathrm{nT}}_{\mathrm{mul}}+\left(3n+2\right)T_{h}$ $+{3\mathrm{nT}}_{\mathrm{mod}}+{2\mathrm{nT}}_{\mathrm{add}}$
Our Scheme	$2T_{\mathrm{mul}}+T_{h}+T_{\mathrm{add}}+3\left(n-1\right)T_{\mathrm{pa}}$	$3T_{\mathrm{mul}}+T_{h}+{2T}_{\mathrm{add}}$ $+\left(n-1\right)T_{\mathrm{pa}}+T_{\mathrm{sub}}$

## Data Availability

The original contributions presented in this study are included in the article. Further inquiries can be directed to the corresponding author.
